# Occupational cold exposure is associated with upper extremity pain

**DOI:** 10.3389/fpain.2023.1063599

**Published:** 2023-05-31

**Authors:** Albin Stjernbrandt, Hans Pettersson, Viktoria Wahlström, Jens Wahlström, Charlotte Lewis

**Affiliations:** Section of Sustainable Health, Department of Public Health and Clinical Medicine, Umeå University, Umeå, Sweden

**Keywords:** cold exposure, lifting, ergonomics, occupational exposure, musculoskeletal pain, upper extremity, Sweden, occupational health

## Abstract

**Background:**

Occupational cold exposure is common in Sweden but potential impacts on musculoskeletal disorders have not been thoroughly investigated. The primary aim of this study was to determine the associations between occupational contact and ambient cooling in relation to pain in the upper extremity.

**Methods:**

In this cross-sectional study, a digital survey was conducted on a population-based sample of women and men between 24 and 76 years of age, living in northern Sweden. Occupational cold exposure, heavy manual handling, work with vibrating tools as well as the presence of upper extremity pain at different sites were subjectively reported. Associations between exposure and outcome were evaluated using multiple binary logistic regression.

**Results:**

The final study sample included 2,089 (54.4%) women and 1,754 men, with a mean age of 56 years. Hand pain was reported by 196 (5.2%), lower arm pain by 144 (3.8%), and upper arm pain by 451 (11.9%). Severe ambient cooling for more than half of the working time was statistically significantly associated with hand pain (OR: 2.30; 95% CI: 1.23–4.29) and upper arm pain (OR: 1.57; 95% CI: 1.00–2.47) but not lower arm pain (OR: 1.87; 95% CI: 0.96–3.65) after adjusting for gender, age, body mass index, current daily smoking, heavy manual handling, and work with vibrating tools.

**Conclusions:**

Occupational cold exposure was statistically significantly associated with hand pain and upper arm pain. Therefore, occupational cold exposure should be recognized as a potential risk factor for musculoskeletal disorders in the upper extremity.

## 1. Introduction

Musculoskeletal disorders (MSDs) in the upper extremities are common complaints in the working population, and one of the main causes of severe pain (1). A recent systematic literature review concluded on different physical work factors that increase the risk for pain in the upper limb (2). Ergonomic factors such as high physical workload, repetitive arm movements, and work with elevated shoulders all increased the risk of shoulder pain. For elbows and lower arms, the risk factors were high physical workload and repetitive arm movements. High physical workload, repetitive hand and wrist movements, and tasks requiring bent or twisted wrist increased the risk of wrist and hand pain. There are also several literature reviews that have documented the impacts of hand-arm vibration exposure on the occurrence of MSDs (3, 4). However, there is yet insufficient evidence to confidently determine the effects of contact and ambient cooling of the upper extremity in relation to development of pain and disability as consequence of MSDs (5). Previous population-based studies in a Scandinavian setting have documented associations between feeling cold at work and reporting long-standing pain in the hand, arm and shoulder, both in cross-sectional (6) and longitudinal analyses (7). Associations between occupational cold exposure and upper extremity pain has also been reported in specific occupational groups such as seafood production workers (8–10) and meat-processing workers (11). However, in these previous studies, it has been difficult to separate physical workload from occupational cold exposure since they often occur in parallel.

Occupational *ambient cold exposure* has been defined as working at a temperature below 10°C (12). Even in relatively mild temperature, wind and moist can impose a general cooling effect on the entire body. C*ontact cooling* on the other hand, occurs when parts of the body are in contact with cold objects or liquids, and this can produce a pronounced local cooling effect which increases the risk of regional symptoms such as local pain and cold injuries, but seldomly affects the overall thermal balance (13). The effects of cooling are subsequently modified by individual factors, including the insulating capacity of clothing, body composition, and physical activity level (13). In Sweden, official statistics report that roughly 21% of men and 11% of women are occupationally exposed to ambient cold for at least one quarter of their working time (14), indicating that such exposure is indeed common and could have a major impact on the health and productivity of the working population.

The primary aim of this study was to determine the associations between occupational contact and ambient cooling in relation to pain in the upper extremity. The secondary aim was to assess the impact of upper extremity pain on work ability and occurrence of sick leave.

## 2. Materials and methods

### 2.1. Study design and setting

This cross-sectional study was based on a digital survey that was sent to women and men between 24 and 76 years of age living in northern Sweden, sampled from the Swedish population register (Statistics Sweden). Individuals were asked to participate through a letter, and one postal reminder was sent out after four weeks to those who had not yet answered to the initial request. The data collection was performed during the late winter (March and April) of 2021. The sampling and response pattern has previously been described in detail (15).

### 2.2. Description of materials

Numerical data were described as mean values and standard deviation (SD), while categorical variables were presented as numbers and valid percentages. Outcome variables were: hand pain (*having aching/pain in the wrist/hands*); lower arm pain (*having aching/pain in the elbow/lower arm*); and upper arm pain (*having aching/pain in the shoulder/upper arm*). Answers were given on four-graded scales ranging from *none* to *a lot*, where *a lot* was considered a positive response. All subjects that did not acknowledge pain to such an extent were considered healthy references. Occupational cold exposure was assessed by three different questionnaire items: contact cooling (*handling of cold objects with a temperature at or below 0°C with the hands*); ambient cooling (*being exposed to cold environments such as outdoor work in the winter, work in refrigerated rooms or similar*); and severe ambient cooling (*being exposed to cold, moisture, and wind that induces cooling despite adequate clothing*). Answers were reported on six-graded time scales ranging from *never* to *almost always*. On the same scale, work with vibrating tools was also asked about (*being exposed to vibration from handheld machines or tools, e.g., a drilling machine*). All six answer categories were used when variables were treated as adjusting covariates in multiple modeling but grouped into three larger categories when used as main independent variables: *never*; *less than half the time*; and *half the time or more*. Occupational physical workload was assessed using an item regarding heavy manual handling (*lifting at least 15 kilograms per unit multiple times per day*), where answers were given on a five-graded time scale, ranging from *never* to *every day*. Additional variables used for adjusting were: *gender (male/female)*; *age (years)*; *body mass index (BMI; kg/m²)*; and *current daily smoking (yes/no)*. Work ability was assessed using the Work Ability Score (WAS) from the Work Ability Index, where the current work ability is subjectively compared to the lifetime best on a whole number numerical rating scale ranging from one to ten (16). Any occurrence of sick leave during the last year was also asked about. Occupation was specified in free-form text and subsequently coded using major and sub-major groups from the International Standard Classification of Occupations (17). Age was categorized into four similar spans, and BMI separated by clinically used thresholds for under- and overweight (18).

### 2.3. Statistical analysis

Statistically significant differences in frequencies between categories were determined using Pearson's chi-squared test, while significance testing for numerical variables was performed using independent samples *t*-test. Correlation between variables was calculated using Spearman's rank correlation coefficient (*r_s_*). For modelling associations between dependent and independent variables, simple (crude) and multiple (adjusted) binary logistic regression was used. A *p* value <0.05 was considered statistically significant. Statistical analyses were performed using IBM SPSS Statistics for Windows (Version 28, IBM Corporation, Armonk, NY, USA).

### 2.4. Ethical considerations

This study was performed in line with the principles of the Declaration of Helsinki. The study protocol was approved by the Swedish Ethical Review Authority (DNR 2020-06707). Written informed consent was obtained from all individual participants included in the study.

## 3. Results

### 3.1. Participants and descriptive data

**Figure 1:**
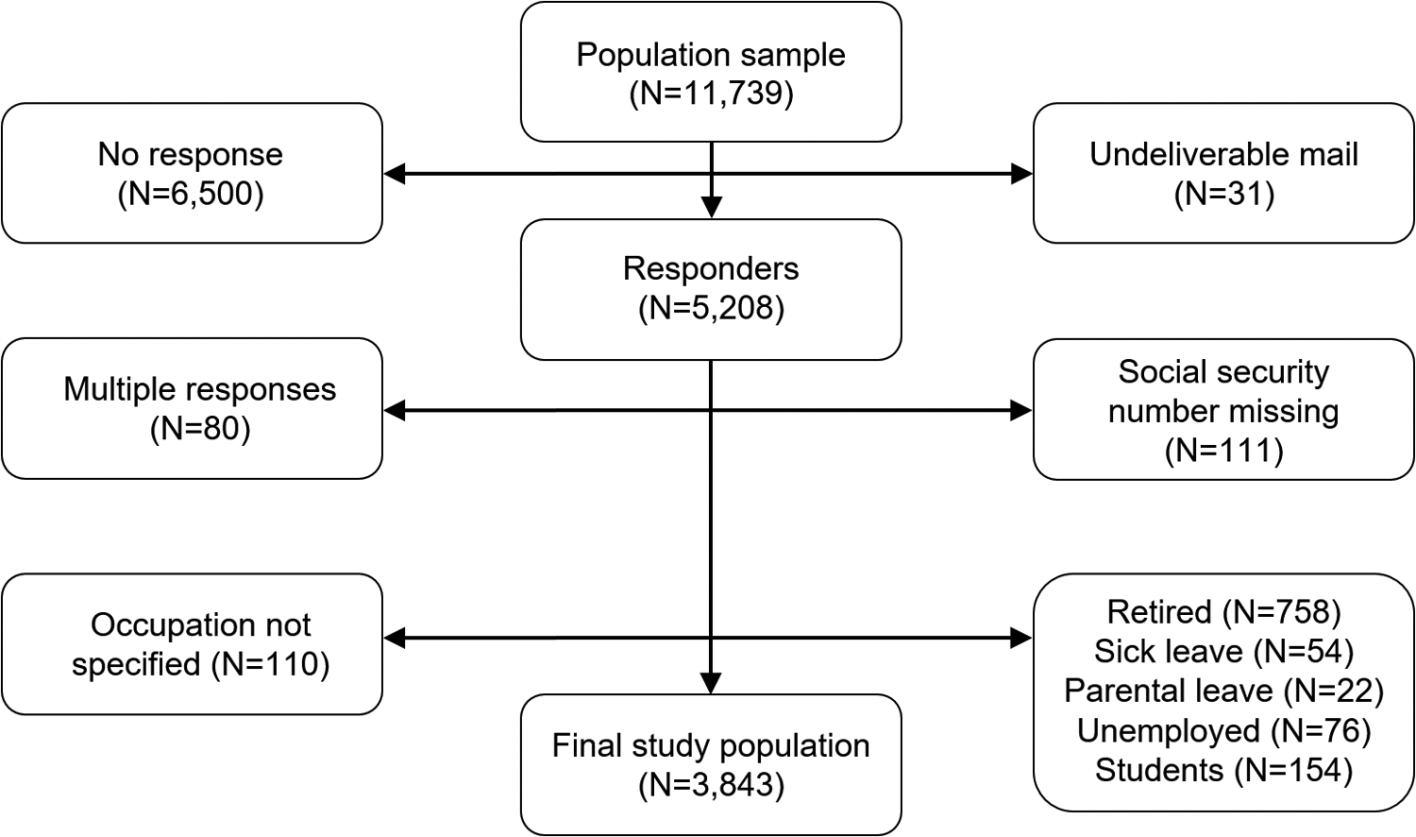
Flow chart showing the data collection for the study.

There were 5,208 responses to the survey, yielding a response rate of 44.4%. After manual review, 191 survey responses could not be used due to multiple or erroneous data entries, leaving 5,017 subjects available for analysis. Since the study focused on occupational exposures, subjects that were retired, unemployed, on sick or parental leave, or students were excluded (*N* = 1,064), as well as those who had not specified their occupation in the survey (*N* = 110) (Figure 1). The final study sample therefore included 2,089 (54.4%) women and 1,754 men, with a mean (SD) age of 56 (11) years. Regarding occupation, 1,093 (28.4%) were professionals, 613 (16.0%) service and sales workers, 594 (15.5%) technicians and associate professionals, 475 (12.4%) clerical support workers, 290 (7.5%) plant and machine operators, 259 (6.7%) managers, 239 (6.2%) crafts workers, 100 (2.6%) self-employed, 98 (2.6%) manual workers, 64 (1.7%) agricultural and fishery workers, and 18 (0.5%) professional militaries.

Hand pain was reported by 196 (5.2%), lower arm pain by 144 (3.8%), and upper arm pain by 451 (11.9%). Any upper extremity pain, i.e., having at least one of the outcomes, was reported by 559 (15.0%). Among those excluded from the original sample due to not working (*N* = 1,174), the occurrence of hand pain was 7.5%, lower arm pain 4.4%, and upper arm pain 13%. The difference in occurrence between the workers (final study sample) and non-workers (excluded participants) was statistically significant for hand pain (*p *= 0.004) but not for lower or upper arm pain (*p *= 0.361 and *p *= 0.350, respectively). Additional descriptive characteristics of the study participants are presented in Table 1 and physical exposures in Table 2.

**Table 1:** Descriptive characteristics of the study participants.

Variable	Categories	Upper extremity pain		Healthy references		*p* value
		*N*	%	*N*	%	
Participants	–	559	15.0	3,173	85.0	
Gender	Female	360	64.4	1,668	52.6	<0.001
	Male	199	35.6	1,505	47.4	
Age (years)	24–37	31	5.5	268	8.4	<0.001
	38–50	85	15.2	706	22.3	
	51–63	270	48.3	1,216	38.3	
	64–76	173	30.9	983	31.0	
Body mass index (kg/m^2^)	BMI < 18.5	6	1.1	21	0.7	<0.001
	18.5 ≤ BMI < 25	179	33.0	1,359	42.8	
	25 ≤ BMI < 30	216	39.9	1,219	39.1	
	BMI ≥ 30	141	26.0	518	16.6	
Current daily smoking	Yes	35	6.4	93	2.9	<0.001
	No	515	93.6	3,060	97.1	

**Table 2:** Descriptive characteristics of the physical exposures.

Variable	Categories	Upper extremity pain		Healthy references		*p* value
		*N*	%	*N*	%	
Contact cooling	Never	336	65.4	2,142	73.6	<0.001
	One tenth of time	100	19.5	495	17.0	
	One quarter of time	32	6.2	132	4.5	
	Half the time	17	3.3	66	2.3	
	Three quarters of time	17	3.3	45	1.5	
	Almost always	12	2.3	31	1.1	
Ambient cooling	Never	291	56.8	1,799	61.6	0.005
	One tenth of time	101	19.7	618	21.2	
	One quarter of time	59	11.5	256	8.8	
	Half the time	28	5.5	143	4.9	
	Three quarters of time	20	3.9	52	1.8	
	Almost always	13	2.5	51	1.7	
Severe ambient cooling	Never	311	61.0	2,019	69.3	<0.001
	One tenth of time	102	20.0	585	20.1	
	One quarter of time	45	8.8	149	5.1	
	Half the time	26	5.1	88	3.0	
	Three quarters of time	15	2.9	41	1.4	
	Almost always	11	2.2	31	1.1	
Heavy manual handling	Never	301	59.0	2,016	69.3	<0.001
	A few days per month	69	13.5	322	11.1	
	One day per week	22	4.3	132	4.5	
	A couple of days per week	47	9.2	208	7.2	
	Every day	71	13.9	229	7.9	
Work with vibrating tools	Never	391	76.2	2,408	82.7	0.013
	One tenth of time	67	13.1	282	9.7	
	One quarter of time	26	5.1	99	3.4	
	Half the time	11	2.1	60	2.1	
	Three quarters of time	10	1.9	31	1.1	
	Almost always	8	1.6	30	1.0	

### 3.2. Cold exposure and upper extremity pain

There were statistically significant associations between occupational cold exposure and upper extremity pain for all three anatomical regions in crude analyses (Table 3). After adjusting for gender, age, BMI, smoking, heavy manual handling, and work with vibrating tools, there were still significant associations between exposure to contact cooling half the time or more and hand pain (OR: 3.04; 95% CI: 1.53–6.06) as well as upper arm pain (OR: 1.63; 95% CI: 1.00–2.67), and between severe ambient cooling half the time or more in relation to hand pain (OR: 2.30; 95% CI: 1.23–4.29) and upper arm pain (OR: 1.57; 95% CI: 1.00–2.47). None of the cold exposure measures were statistically significantly associated with lower arm pain in the fully adjusted model. Gender-stratified results are available in (Supplementary Tables S4, S5). There was statistically significant covariance between heavy manual handling and contact cooling (*r_s_*_ _= 0.54; *p* < 0.001), ambient cooling (*r_s_*_ _= 0.49; *p* < 0.001), and severe ambient cooling (*r_s_*_ _= 0.47; *p* < 0.001). There was also significant covariance between work with vibrating tools and contact cooling (*r_s_*_ _= 0.54; *p* < 0.001), ambient cooling (*r_s_*_ _= 0.44; *p* < 0.001), and severe ambient cooling (*r_s_*_ _= 0.44; *p* < 0.001).

**Table 3:** Binary logistic regression for occupational cold exposure in relation to upper extremity pain.

Exposure variable	Exposure level (proportion of working hours)	Hand pain				Lower arm pain				Upper arm pain			
		Crude	Model 1	Model 2	Model 3	Crude	Model 1	Model 2	Model 3	Crude	Model 1	Model 2	Model 3
		OR (95% CI)	OR (95% CI)	OR (95% CI)	OR (95% CI)	OR (95% CI)	OR (95% CI)	OR (95% CI)	OR (95% CI)	OR (95% CI)	OR (95% CI)	OR (95% CI)	OR (95% CI)
Contact cooling	Never	1 (−)	1 (−)	1 (−)	1 (−)	1 (−)	1 (−)	1 (−)	1 (−)	1 (−)	1 (−)	1 (−)	1 (−)
	Less than half the time	1.26 (0.88–1.81)	**1.70** (**1.15–2.53)**	1.46 (0.94–2.25)	1.41 (0.90–2.20)	1.42 (0.96–2.10)	**1.77 (1.16–2.69**	1.37 (0.86–2.17)	1.31 (0.81–2.11)	**1.51** (**1.20–1.91)**	**1.92** (**1.49–2.47)**	**1.57** (**1.19–2.09)**	**1.47** (**1.10–1.97)**
	Half the time or more	**2.21** (**1.31–3.73)**	**3.82** (**2.18–6.69)**	**2.96** (**1.58–5.57)**	**3.04** (**1.53–6.06)**	**2.00** (**1.10–3.65)**	**2.69** (**1.43–5.08)**	1.79 (0.88–3.61)	1.81 (0.85–3.87)	**1.96** (**1.33–2.87)**	**2.65** (**1.75–4.00)**	**1.88** (**1.20–2.97)**	**1.63** (**1.00–2.67)**
Ambient cooling	Never	1 (−)	1 (−)	1 (−)	1 (−)	1 (−)	1 (−)	1 (−)	1 (−)	1 (−)	1 (−)	1 (−)	1 (−)
	Less than half the time	1.32 (0.94–1.84)	**1.70** (**1.19–2.43)**	1.44 (0.98–2.11)	1.40 (0.95–2.06)	1.18 (0.81–1.72)	1.45 (0.97–2.17)	1.17 (0.76–1.80)	1.13 (0.73–1.75)	1.16 (0.93–1.46)	**1.43** (**1.12–1.82)**	1.19 (0.92–1.55)	1.14 (0.87–1.49)
	Half the time or more	1.50 (0.91–2.47)	**2.54** (**1.49–4.33)**	**1.86** (**1.04–3.35)**	1.76 (0.96–3.23)	**1.75** (**1.04–2.94)**	**2.45** (**1.41–4.27)**	1.67 (0.90–3.08)	1.60 (0.85–3.00)	**1.59** (**1.14–2.20)**	**2.24** (**1.57–3.19)**	**1.55** (**1.05–2.29)**	1.36 (0.90–2.04)
Severe ambient cooling	Never	1 (−)	1 (−)	1 (−)	1 (−)	1 (−)	1 (−)	1 (−)	1 (−)	1 (−)	1 (−)	1 (−)	1 (−)
	Less than half the time	1.17 (0.83–1.67)	**1.57** (**1.07–2.29)**	1.29 (0.86–1.94)	1.27 (0.84–1.91)	1.20 (0.81–1.78)	**1.51** (**1.00–2.29)**	1.20 (0.77–1.86)	1.17 (0.75–1.83)	**1.44** (**1.15–1.81)**	**1.84** (**1.44–2.35)**	**1.55** (**1.20–2.02)**	**1.50** (**1.15–1.97)**
	Half the time or more	**2.20** (**1.34–3.63)**	**3.09** (**1.78–5.36)**	**2.32** (**1.28–4.21)**	**2.30** (**1.23–4.29)**	**2.22** (**1.27–3.86)**	**2.71** (**1.49–4.94)**	1.85 (0.97–3.53)	1.87 (0.96–3.65)	**1.90** (**1.31–2.76)**	**2.48** (**1.66–3.69)**	**1.78** (**1.16–2.74)**	**1.57** (**1.00–2.47)**

OR, odds ratio, 95% CI: ninety-five percent confidence interval.

Bold values indicate odds ratios with significant 95% confidence intervals.

Model 1: Adjusted for gender, age, body mass index, and current daily smoking.

Model 2: Model 1 also adjusted for heavy manual handling.

Model 3: Model 2 also adjusted for work with vibrating tools.

### 3.3. Upper extremity pain and work ability

The mean (SD) WAS was 6.58 (2.67) for subjects with hand pain, 7.26 (2.43) for those with lower arm pain, and 7.02 (2.43) for those with upper arm pain, compared to 8.21 (1.95) among those without any upper extremity pain (*p *< 0.001 for all three pain locations). Any occurrence of sick leave during the last year was reported by 62 (32.3%) of subjects with hand pain, 46 (32.2%) of those with lower arm pain, and 117 (26.3%) of those with shoulder pain, compared to 546 (17.6%) among healthy references (*p *< 0.001 for all three pain locations).

## 4. Discussion

### 4.1. Key findings

This population-based study showed statistically significant associations between occupational cold exposure and pain in different parts of the upper extremity. The strongest associations were found between contact cooling and hand pain. The work ability was lower and occurrence of recent sick leave higher among those who reported upper extremity pain than among healthy references.

### 4.2. Interpretation

In our study, any occurrence of upper extremity pain (regardless of location) was reported by 15%, which can be compared to official Swedish statistics, where roughly 19% reported work-related issues in the neck, shoulder and arm (19). In a recent scoping review, it was concluded that studies on the association between cold exposure and MSDs are heterogenous with regards to study samples, measures of exposure and outcome, as well as methodological approaches (5). However, the included studies generally indicated that cold exposure increases the risk of MSDs, and this was demonstrated separately for the hands and wrists (10, 11, 20, 21), lower arms and elbows (11, 20, 21) as well as upper arms and shoulders (10, 20, 22, 23). The effect sizes in the included studies varied widely, with point estimates (OR) ranging from 1.1 to 20.1, reflecting the heterogenous nature of the previous literature (5). In the study by Farbu et al., which was conducted in a quite similar setting as our study (as a population-based cohort in northern Norway), chronic pain in the hands was reported by 6%, in the arms 8%, and in the shoulders 13% among those who worked in a cold environment less than one quarter of the time (6). For those who reported a more frequent exposure and were also often feeling cold at work, the prevalence for each anatomical location was higher (12%, 21%, and 35%, respectively). In comparison to the low-exposed subjects in the study by Farbu et al., our study sample had a similar occurrence of hand or wrist pain (5%) as well as upper arm or shoulder pain (12%), but a much lower estimate of lower arm or elbow pain (3.8%). However, we lacked data on duration of pain and had broader anatomical region of interest, which may partly explain the difference in occurrence. In the study by Farbu et al., working in a cold environment for more than a quarter of the time was associated with hand pain (OR: 1.16; 95% CI: 0.79–1.71), arm pain (OR: 1.34; 95% CI: 0.98–1.83), and shoulder pain (OR: 1.39; 95% CI: 1.08–1.78), after adjustment for gender, age, educational level, body mass index, insomnia, physical activity at work and leisure-time, and smoking. In comparison, our study generally showed rather similar effect estimates, where severe ambient cooling for half the working time or more was associated with hand pain (OR: 2.30; 95% CI: 1.23–4.29), lower arm pain (OR: 1.87; 95% CI: 0.96–3.65), and upper arm pain (OR: 1.57; 95% CI: 1.00–2.47), after adjusting for gender, age, BMI, smoking, heavy manual handling, and work with vibrating tools. It is likely that adding more covariates to our model would have attenuated the associations further, making it even more similar to the study by Farbu et al. There was also a difference in the categorization of exposure in the studies, which could also influence the effect size estimates. To conclude, the occurrence of upper extremity pain in our study was roughly in line with other recent investigations, although temporal data and more specific definitions of pain outcomes would have made comparisons easier. Our findings of statistical associations between contact and ambient cooling in relation to reporting upper extremity pain are also in line with the general trend of previous research (5).

Interestingly, in our study, the point estimate for hand pain was higher for contact cooling than ambient cooling or severe ambient cooling. This fact suggests a strong local effect where heat is transferred away from the tissues of the hands due to contact with cold objects, such as tools or goods. It has previously been shown that conductive heat loss is very efficient compared to convective, evaporative, and radiative heat loss (24). This is especially true if the hand is in contact with metal surfaces (25). The cooling effect is also modified by the grip force and the potential use of gloves (26). Further, in our study, the effect sizes were generally larger for severe ambient cooling (i.e., with the addition of wind, moisture, or insufficient clothing) compared to ambient cooling of a more modest degree. Although the ratings of cold exposure on these scales were subjective, it is reasonable to assume that the cooling effect was more pronounced in the former exposure item than in the latter. This view is supported by a previous review, where outdoor working conditions that involve sudden temperature changes, wind, and precipitation were considered to exert a more pronounced cooling effect and could also complicate the utilization of appropriate cold protection such as jackets, hats, and gloves (13).

Previous studies have described heavy manual handling as well as work with vibrating tools to be independent risk factors of pain in the hands (27, 28), elbows (4), and shoulders (3). In our study, both these physical exposures were strongly correlated with the degree of occupational cold exposure (with correlation coefficients ranging between 0.44–0.54), making it hard to separate effects in logistic regression modelling. However, the statistically significant associations between the three different occupational cold exposure measures and the upper extremity pain outcomes that were estimated in crude analyses were not entirely neutralized when adjusting for heavy manual handling and work with vibrating tools in the final model (Table 3), suggesting an added effect of cold exposure. In this context, it should be clearly stated that the effects of occupational cold exposure on lower arm pain were not statistically significant in the final model. It is therefore possible that heavy manual handling and work with vibrating tools were stronger predictors of lower arm pain than cold exposure, and that the added effect of cooling was negligible for this outcome. Importantly, work with vibrating tools does not only involve hand-transmitted vibration *per se*, but also exposure to forceful gripping and other means of biomechanical loading of the upper extremity (29). Since the majority of vibration from hand tools is deposited in the fingers and hand, and only some low-frequency content propagated to the upper arm and shoulder, it is unlikely that direct mechanical injury from vibration is the cause of pain in the more proximal parts of the upper extremity (30), but rather a consequence of the other ergonomic factors.

The mechanisms behind cold-related MSDs are not entirely understood. Most obvious, cooling of tissues can induce pain on its own, through the activation of transient receptor potential channels that convey afferent sensory information regarding noxious cold stimuli (31). In this context, cold-induced peripheral and central sensitization is also believed to augment pain responses (32). In studies on military personnel, it has been shown that intense cold exposure can be followed by cold allodynia and abnormal thresholds for thermal quantitative sensory testing in the hands (33). These effects are likely due to alteration of both large- and small-fiber nerve function (34). In addition, cold exposure can reduce the biomechanical integrity of connective tissue, increasing the risk of sprains and ligamental tears (35). Cooling of skeletal muscles can increase the muscular tone at rest and reduce the contractive force, increasing the risk of fatigue, ischemia, and myalgia (36, 37). Cold exposure can also cause discomfort and change the perception of pain due to emotional modulation (38).

### 4.3. Limitations

There were several limitations in our study. First of all, there was a large proportion of non-responders, which may have affected the generalizability of the results. By excluding non-working subjects, we may have induced a healthy worker effect that could have attenuated the associations between occupational exposures and the outcomes. This could be suspected based on the significantly higher frequency of hand pain among non-working subjects that were subsequently not included in further analyses. Secondly, because of the cross-sectional design, it cannot be concluded if cold exposure contributed to upper extremity pain or if workers with such conditions were more prone to report a high exposure. Moreover, it would have been valuable to have more detailed information on the duration and distribution of pain, as well as diagnoses established by the healthcare. However, since the work ability was significantly lower among those who reported upper extremity pain in our study, and the proportion of sick leave higher, it is reasonable to assume that the pain outcomes were not completely negligible or transient in nature. The exposure data on ergonomic factors could also have been more detailed, including hand-intensive work tasks requiring grip force and repetitive finger or wrist movements. The covariance between the cold exposure variables and heavy manual handling as well as work with vibrating tools made it difficult to establish the independent effect of contact and ambient cooling on the outcomes. Moreover, our study did not include leisure-time cold exposure, which might have had an influence on pain occurrence. Finally, our study did not collect data on concurrent conditions such as vascular disease, diabetes mellitus or mood disorders, which means that any effect of such conditions on the pain outcomes could not be investigated. Thus, the results of our study should mainly be considered hypotheses-generating, to be confirmed by well-controlled prospective studies with validated measures of both exposure and outcome. Future studies on this topic should preferably also include instruments on mental health and consider emotional modulation of pain.

### 4.4. Strengths

However, there were also inherent strengths in our study. The sample size was large and population-based, using the national population register for randomized selection of potential study participants. The study sample mainly consisted of working-age subjects that lived in a cold climate and had a broad range of occupations, which means that the study likely captures an average effect of cold exposure on the general working population. Careful adjustments of the binary logistic regression models could be made, using covariates that previously have been established as factors that affect the reporting of upper extremity pain. Therefore, we believe that our results are valid and give a broad indication of the potential effect of cold exposure in relation to upper extremity pain. Cold exposure among outdoor workers in the north may be an overlooked occupational hazard that deserves more attention from an occupational health and safety perspective. There is a standard from the International Organization for Standardization (15743:2008) that describes cold risk assessment and management, both from a technical and medical perspective. This standard has seen little use in Scandinavian countries, and a broader implementation could be beneficial for workers’ health.

## 5. Conclusions

Occupational cold exposure was statistically significantly associated with hand pain and upper arm pain. Therefore, occupational cold exposure should be recognized as a potential risk factor for musculoskeletal disorders in the upper extremity.

## Data Availability

The raw data supporting the conclusions of this article will be made available by the authors, without undue reservation.
