# Supplementary material for: Occupational cold exposure is associated with upper extremity pain
Source: Front Pain Res (Lausanne). 2023 May 31;4:1063599. doi: 10.3389/fpain.2023.1063599 (PMC10264621; doi:10.3389/fpain.2023.1063599)
Supplement: Supplementary file 1 [file Datasheet1.pdf]

Table 4. Binary logistic regression for occupational cold exposure in relation to upper extremity pain for women (N=2,089)

**Exposure information**		**Hand pain**				**Lower arm pain**				**Upper arm pain**			
Variable	Duration	Crude	Model 1	Model 2	Model 3	Crude	Model 1	Model 2	Model 3	Crude	Model 1	Model 2	Model 3
		OR (95% CI)	OR (95% CI)	OR (95% CI)	OR (95% CI)	OR (95% CI)	OR (95% CI)	OR (95% CI)	OR (95% CI)	OR (95% CI)	OR (95% CI)	OR (95% CI)	OR (95% CI)
Contact cooling	Never	1 (-)	1 (-)	1 (-)	1 (-)	1 (-)	1 (-)	1 (-)	1 (-)	1 (-)	1 (-)	1 (-)	1 (-)
	Less than half the time	**1.86 (1.16–2.99)**	**1.77 (1.09–2.90)**	1.56 (0.93–2.63)	1.50 (0.88–2.57)	1.36 (0.76–2.46)	1.35 (0.74–2.48)	1.22 (0.64–2.30)	1.10 (0.57–2.11)	**1.79 (1.27–2.54)**	**1.84 (1.29–2.63)**	**1.59 (1.08–2.32)**	**1.59 (1.08–2.34)**
	Half the time or more	**3.71 (1.67–8.23)**	**4.41 (1.94–10.05)**	**3.43 (1.39–8.45)**	**3.74 (1.39–10.08)**	**3.38 (1.38–8.27)**	**3.36 (1.34–8.44)**	2.55 (0.92–7.05)	2.02 (0.65–6.27)	1.97 (0.96–4.04)	1.88 (0.87–4.08)	1.42 (0.62–3.23)	1.36 (0.55–3.40)
Ambient cooling	Never	1 (-)	1 (-)	1 (-)	1 (-)	1 (-)	1 (-)	1 (-)	1 (-)	1 (-)	1 (-)	1 (-)	1 (-)
	Less than half the time	**1.57 (1.04–2.38)**	**1.64 (1.07–2.53)**	1.48 (0.95–2.33)	1.50 (0.95–2.36)	1.30 (0.79–2.12)	1.45 (0.87–2.41)	1.33 (0.78–2.26)	1.29 (0.75–2.20)	1.14 (0.83–1.56)	1.27 (0.92–1.75)	1.15 (0.82–1.61)	1.18 (0.84–1.65)
	Half the time or more	**2.56 (1.26–5.17)**	**2.95 (1.42–6.13)**	**2.31 (1.06–5.03)**	**2.38 (1.08–5.23)**	**2.64 (1.21–5.74)**	**2.82 (1.26–6.34)**	2.35 (0.99–5.57)	2.08 (0.85–5.09)	**1.90 (1.08–3.33)**	**2.21 (1.23–3.96)**	1.73 (0.93–3.21)	1.68 (0.89–3.19)
Severe ambient cooling	Never	1 (-)	1 (-)	1 (-)	1 (-)	1 (-)	1 (-)	1 (-)	1 (-)	1 (-)	1 (-)	1 (-)	1 (-)
	Less than half the time	**1.59 (1.01–2.50)**	**1.60 (1.00–2.55)**	1.43 (0.88–2.33)	1.43 (0.88–2.34)	1.22 (0.70–2.14)	1.27 (0.71–2.25)	1.15 (0.63–2.07)	1.14 (0.63–2.07)	**1.51 (1.09–2.09)**	**1.62 (1.16–2.27)**	**1.46 (1.03–2.08)**	**1.50 (1.05–2.13)**
	Half the time or more	**3.61 (1.81–7.18)**	**3.85 (1.83–8.06)**	**3.15 (1.44–6.87)**	**3.31 (1.47–7.44)**	**3.36 (1.53–7.37)**	**3.65 (1.62–8.24)**	**2.93 (1.24–6.96)**	**2.71 (1.10–6.72)**	1.81 (0.97–3.41)	**1.96 (1.00–3.83)**	1.57 (0.78–3.16)	1.54 (0.73–3.21)

OR odds ratio, *95% CI* ninety-five percent confidence interval

Bold values indicate odds ratios with significant 95% confidence intervals

Model 1: Adjusted for age, body mass index, and current daily smoking

Model 2: Model 1 also adjusted for heavy manual handling

Model 3: Model 2 also adjusted for work with vibrating tools

Table 5. Binary logistic regression for occupational cold exposure in relation to upper extremity pain for men (N=1,754)

**Exposure information**		**Hand pain**				**Lower arm pain**				**Upper arm pain**			
Variable	Duration	Crude<br>OR (95% CI)	Model 1<br>OR (95% CI)	Model 2<br>OR (95% CI)	Model 3<br>OR (95% CI)	Crude<br>OR (95% CI)	Model 1<br>OR (95% CI)	Model 2<br>OR (95% CI)	Model 3<br>OR (95% CI)	Crude<br>OR (95% CI)	Model 1<br>OR (95% CI)	Model 2<br>OR (95% CI)	Model 3<br>OR (95% CI)
Contact cooling	Never	1 (-)	1 (-)	1 (-)	1 (-)	1 (-)	1 (-)	1 (-)	1 (-)	1 (-)	1 (-)	1 (-)	1 (-)
	Less than half the time	1.60 (0.85–3.00)	1.49 (0.77–2.87)	1.28 (0.59–2.76)	1.13 (0.50–2.57)	**2.38 (1.28–4.43)**	**2.27 (1.21–4.25)**	1.39 (0.67–2.88)	1.61 (0.76–3.41)	**2.02 (1.41–2.89)**	**2.01 (1.40–2.90)**	**1.56 (1.01–2.38)**	1.35 (0.86–2.12)
	Half the time or more	**3.15 (1.45–6.83)**	**3.27 (1.50–7.16)**	**2.69 (1.03–7.03)**	2.48 (0.86–7.15)	**2.54 (1.04–6.19)**	**2.51 (1.03–6.13)**	1.28 (0.46–3.57)	1.97 (0.66–5.94)	**2.98 (1.82–4.88)**	**3.06 (1.85–5.06)**	**2.05 (1.14–3.70)**	1.72 (0.90–3.28)
Ambient cooling	Never	1 (-)	1 (-)	1 (-)	1 (-)	1 (-)	1 (-)	1 (-)	1 (-)	1 (-)	1 (-)	1 (-)	1 (-)
	Less than half the time	1.83 (0.96–3.47)	1.80 (0.92–3.52)	1.38 (0.65–2.90)	1.24 (0.58–2.66)	1.50 (0.78–2.86)	1.41 (0.73–2.72)	0.87 (0.42–1.81)	0.93 (0.45–1.95)	**1.66 (1.14–2.40)**	**1.64 (1.12–2.40)**	1.23 (0.81–1.87)	1.12 (0.73–1.73)
	Half the time or more	2.07 (0.93–4.62)	2.21 (0.98–5.00)	1.54 (0.59–4.04)	1.32 (0.48–3.62)	2.14 (0.99–4.64)	2.12 (0.98–4.61)	1.00 (0.41–2.46)	1.24 (0.49–3.15)	**2.25 (1.42–3.55)**	**2.32 (1.46–3.70)**	1.40 (1.81–2.41)	1.17 (0.66–2.07)
Severe ambient cooling	Never	1 (-)	1 (-)	1 (-)	1 (-)	1 (-)	1 (-)	1 (-)	1 (-)	1 (-)	1 (-)	1 (-)	1 (-)
	Less than half the time	1.43 (0.77–2.66)	1.43 (0.76–2.71)	1.05 (0.51–2.17)	0.95 (0.45–2.00)	1.75 (0.94–3.26)	1.75 (0.94–3.27)	1.12 (0.56–2.24)	1.23 (0.61–2.49)	**2.08 (1.45–2.99)**	**2.13 (1.47–3.08)**	**1.68 (1.11–2.53)**	**1.55 (1.02–2.36)**
	Half the time or more	**2.58 (1.16–5.75)**	**2.39 (1.03–5.57)**	1.64 (0.62–4.34)	1.44 (0.51–4.06)	**2.52 (1.08–5.87)**	2.16 (0.89–5.26)	1.09 (0.40–2.94)	1.48 (0.53–4.12)	**3.00 (1.82–4.94)**	**2.94 (1.76–4.93)**	**1.95 (1.08–3.50)**	1.67 (0.90–3.09)

OR odds ratio, *95% CI* ninety-five percent confidence interval

Bold values indicate odds ratios with significant 95% confidence intervals

Model 1: Adjusted for age, body mass index, and current daily smoking

Model 2: Model 1 also adjusted for heavy manual handling

Model 3: Model 2 also adjusted for work with vibrating tools
